# Climate-Driven Phenological Change: Developing Robust Spatiotemporal Modeling and Projection Capability

**DOI:** 10.1371/journal.pone.0141207

**Published:** 2015-11-06

**Authors:** Carmen Prieto, Georgia Destouni

**Affiliations:** Department of Physical Geography, Bolin Centre for Climate Research, Stockholm University, Stockholm, Sweden; University of Thessaly, GREECE

## Abstract

Our possibility to appropriately detect, interpret and respond to climate-driven phenological changes depends on our ability to model and predict the changes. This ability may be hampered by non-linearity in climate-phenological relations, and by spatiotemporal variability and scale mismatches of climate and phenological data. A modeling methodology capable of handling such complexities can be a powerful tool for phenological change projection. Here we develop such a methodology using citizen scientists’ observations of first flight dates for orange tip butterflies (*Anthocharis cardamines*) in three areas extending along a steep climate gradient. The developed methodology links point data of first flight observations to calculated cumulative degree-days until first flight based on gridded temperature data. Using this methodology we identify and quantify a first flight model that is consistent across different regions, data support scales and assumptions of subgrid variability and observation bias. Model application to observed warming over the past 60 years demonstrates the model usefulness for assessment of climate-driven first flight change. The cross-regional consistency of the model implies predictive capability for future changes, and calls for further application and testing of analogous modeling approaches to other species, phenological variables and parts of the world.

## Introduction

Phenological changes due to climate change have the potential to negatively affect species conservation and interactions, and may result in decrease of biodiversity [1]. Reliable predictions of phenological changes may therefore be essential for ecosystem protection and management. Predictions of phenological change due to climate change are often based on models that relate observed changes in some phenological event with some phenologically decoupled measure of climate change. The latter may be expressed as change in average temperature [2,3,4] or in cumulative degree-days (DD) over a fixed time period at some geographical location [5,6]. However, fundamental non-linearity emerges from the fact that the relevant averaging time or accumulation period for the climate variable (temperature or DD) is not known a priori and depends on the phenological variable under study. For instance, first flight dates (FF) for insects should be related to DD accumulation until FF, rather than until some independent fixed time. This implies that the relevant accumulation time for DD changes along with the climate-driven FF change for a species. Such non-linearity may violate assumptions of independence between driving climate variables and predicted phenological events [7].

Furthermore, the observational data that constitute the basis for phenological change models may include temporal and spatial scale mismatches. For instance, available FF data may apply to some spatiotemporal points, whereas temperature and DD data may apply to other points in a considered area (for direct temperature measurements) or to spatial averages with some resolution (for gridded temperature data). Spatiotemporal variability in data with mismatched support scales may obscure overall relations between climate and phenology, as well as obscure possibilities to generalize relations across different geographical locations and times. There is therefore a need to develop modeling frameworks that appropriately link spatiotemporally variable climate and phenological data with different support scales, as well as account for non-linear dependencies between climate and phenological change variables.

In this study, we address these needs by developing a methodology to modeling climate-driven FF change that accounts for the non-linearity in FF dependence on cumulative DD until FF, and links associated spatiotemporally variable climate and phenological data with different data support scales. The methodology is concretized by application to FF dates of orange tip butterflies (*Anthocharis cardamines*) and includes comparison between different modeling approaches and identification of a best performing model in relation to observed data. Furthermore, the methodology application includes model use for interpreting and understanding long-term climate-driven FF change over the last 60 years in three Swedish regions along a steep climate gradient.

## Materials and Methods

### Observational data

**Fig 1:**
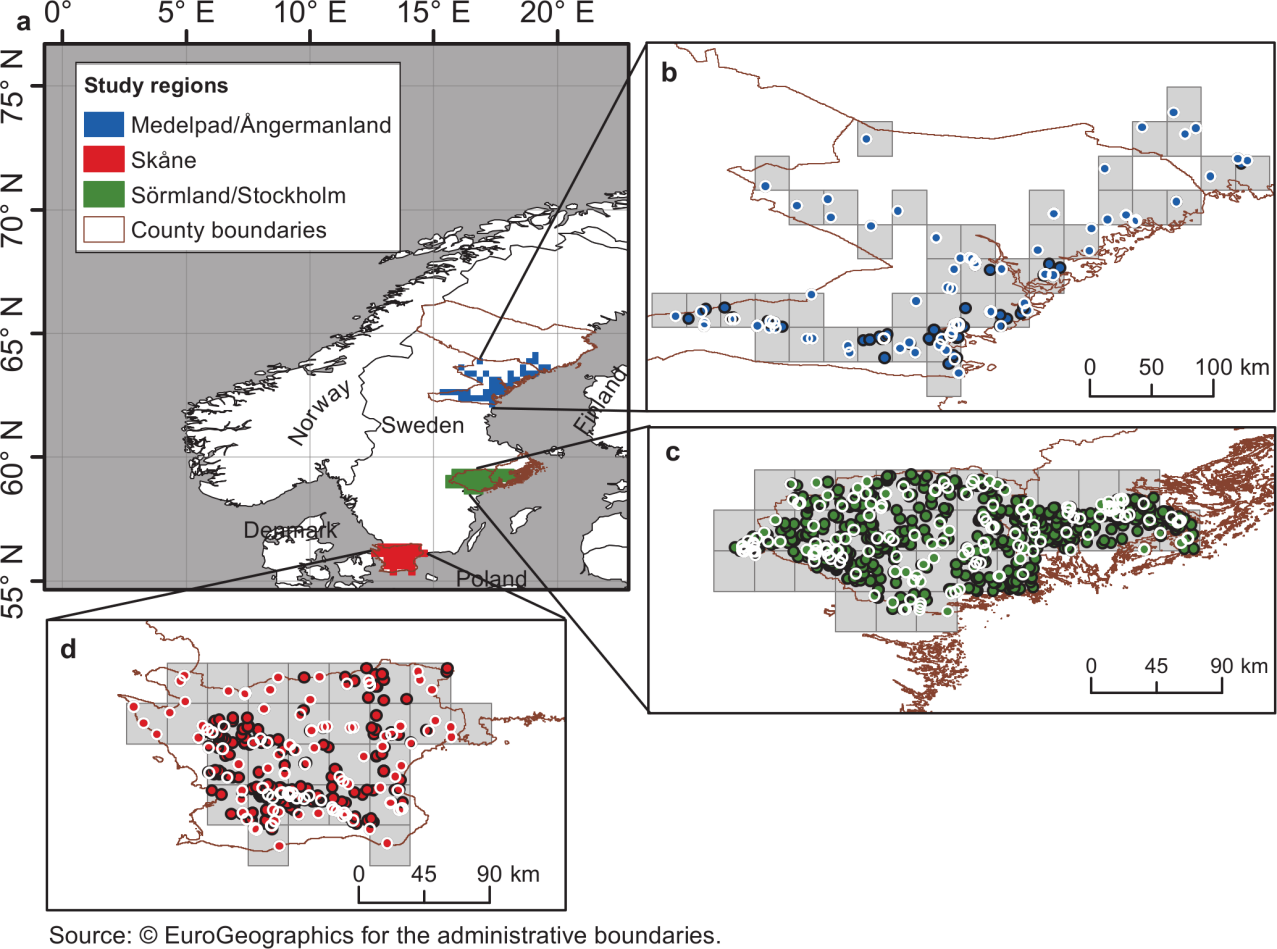
Investigated regions, temperature grid cells and sighting data points. a) Location and extent of the three study regions in Sweden. b-d) Spatial distribution of first flight (FF) sighting points (circles) within each temperature grid cell (gray squares) for the regions: b) Medelpad/Ångermanland, c) Sörmland/Stockholm and d) Skåne. All sighting data (all circles) for all years with such data are considered as FF data under the spatial variability assumption (SVA). Only the white-circled data points, with each representing an earliest sighting for at least one year with data, are considered as FF data under the observation bias assumption (OBA).

For the three study regions in Sweden (Fig 1A), we use reported field observations of orange tip butterflies (*Anthocharis cardamines*; Fig 1B–1D). Observation data are available continuously for the years 2003–2010 in the citizen scientist database Artportalen [8], along with additional data sporadically available for some earlier years. This database provides date and species observed at different spatially referenced locations within the study areas. Temperature data is considered from the E-OBS European dataset [9] for the period 1950–2010 in terms of daily maximum (T_max_) and minimum (T_min_) gridded data values. In particular, we use version 5.0 of the blended data interpolated on a regular grid with cell sizes of 0.25°long × 0.25°lat (gray squares in Fig 1B–1D). According to Haylock et al. [9], this interpolation was carried out by a three step methodology including an initial homogenization of the observed daily station data and the use of a kriging method that was selected to be the best for interpolation of daily anomalies.

Phenological data that does not overlap in space and time with the extent of temperature data within the study areas is not included in the present analysis. For sightings reported on different days at the same location in the same season/year, only the earliest entry for every year is considered as the FF for that year and specific location.

Furthermore, for each temperature grid cell, several different sightings of FF dates may be reported for the same year at different locations within the cell. This variability of FF sightings within each temperature cell may then represent small-scale variability of FF due to actually existing small-scale temperature variability [10,11], or it may represent FF observation bias due to sighting delays, or some combination of both. We therefore investigate the implications of making two contrasting assumptions regarding the intra-cell FF sighting variability for each year. One of these contrasting assumptions is that all of this intra-cell variability depends on actual small-scale spatial variability of local temperature within each grid cell; this is referred to as the spatial variability assumption (SVA). The other assumption is that all of the intra-cell sighting variability depends on observation bias; this is referred to as the observation bias assumption (OBA).

More specifically, in the SVA case, we assume that all sighting dates reported for each year at different spatial locations within each temperature grid cell are correct spatially variable FF dates within the cell. In the OBA case, we consider instead the earliest reported sighting for each year in each temperature grid cell as being the only correct FF date for that year and grid cell. For each grid cell, both the spatial variability of FF sightings in each year and the temporal variability of sightings among different years are thus accounted for in the SVA case (including all data points in Fig 1B–1D), while only the latter, temporal variability of the earliest annual sighting in each grid cell is considered in the OBA case (including only the white-circled points of earliest annual sightings in Fig 1B–1D).

### Modeling methodology

In order to model the variation of FF dates for an insect species, spatially within and among regions and temporally as climate changes in a region, we hypothesize the existence of a species-specific but climate- and region-independent value (DD_C_) of cumulative DD that must on average be achieved until an insect is ready for flight. That is, we assume that this DD_C_ value is primarily determined by physiological development requirements of the insect species that may remain essentially similar across geographic locations and climate conditions. If such a species-specific DD_C_ value can be found based on data across different regions, which is independent of the regional geography and climate, this DD_C_ value should then also be independent of temporal climate change and thereby relevant and useful for modeling climate-driven FF change in any region of interest.

**Fig 2:**
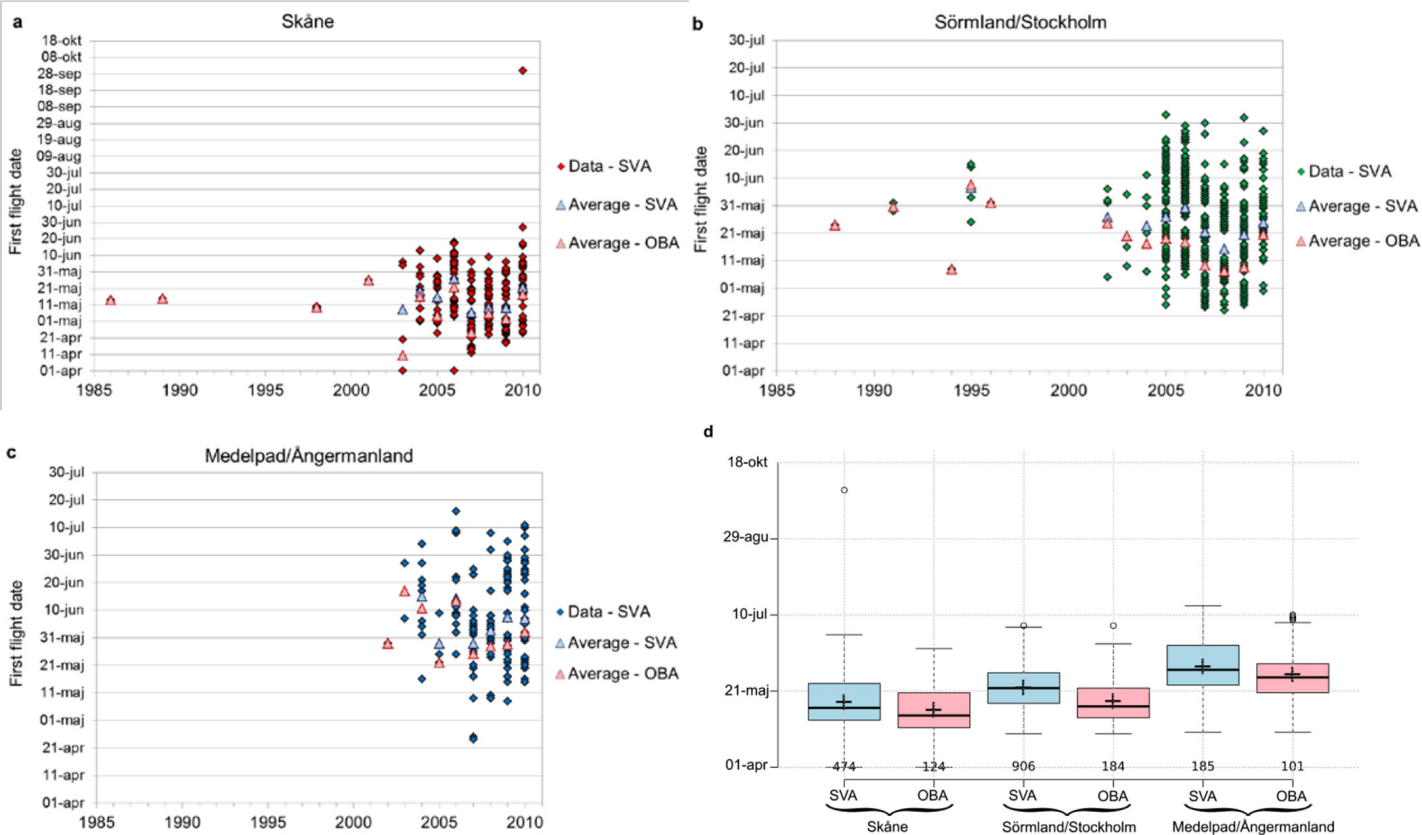
Data and statistics of first flight dates (FF). a-c) FF data for each year shown for the regions: a) Skåne, b) Sörmland/Stockholm and c) Medelpad/Ångermanland. The data shown in panels a-c includes all sighting data within and across all grid cells, as considered in the spatial variability assumption (SVA) case, and their total annual average value (Average–SVA) compared with the annual average value across all cells of the earliest sighting in each grid cell (Average–OBA) as considered in the observation bias assumption (OBA) case (S1 Fig shows additional details of the spatiotemporal variability of FF data). d) Tukey-style box plot of the FF data considered in the SVA and OBA cases for the three investigated regions with means (crosses) and n values. The upper and lower bars show the Tukey–whiskers, which extend to data points of ± 1.5 x IQR, where IQR is the interquartile range.

Under the present DD_C_ hypothesis, it is the annual timing of DD_C_ achievement and not the DD_C_ value itself that should depend on the regional climate conditions. In other words, the first flight date of an insect, FF(DD_C_), should be the time of DD_C_ achievement in each specific insect location and year, and FF(DD_C_) should vary across regions and years mainly due to the variability of the climatic conditions that determine the timing of this DD_C_ achievement. A relevant FF(DD_C_) model must thus be able to explain and reproduce most of the spatiotemporal variability of actually observed FF data (Fig 2, Table 1, S1 Fig) based only on the observed spatiotemporal variability of regional climate conditions. The present study uses observation data of FF and climate to test this ability and thereby the relevance of the FF(DD_C_) model assumption of a species-specific but climate/region-independent average DD_C_ value that must be achieved in order for the insects to be able to fly.

**Table 1:** Summary statistics of observed first flight dates (FF).

	SVA assumption		OBA assumption	
	Mean	SD (days)	Mean	SD (days)
**Spatiotemporal statistics of all FF data**	*Spatiotemporal mean*	*Spatiotempo-ral SD*	*Spatiotemporal mean*	*Spatiotempo-ral SD*
Medelpad/Ångermanland	June 6	16.2	May 31	16.1
Sörmland/Stockholm	May 23	13.9	May 14	14
Skåne	May 13	16.5	May 8	16.6
**Spatial statistics of long-term average FF in each pixel**	*Spatial mean*	*Spatial SD*	*Spatial mean*	*Spatial SD*
Medelpad/Ångermanland	June 8	13.4	June 5	15
Sörmland/Stockholm	May 23	6.3	May 15	9.6
Skåne	May 15	9.1	May 11	10.7
**Temporal statistics of regional average FF in each year**	*Temporal mean*	*Temporal SD*	*Temporal mean*	*Temporal SD*
Medelpad/Ångermanland	June 6	7.7	June 2	9.2
Sörmland/Stockholm	May 24	7.4	May 19	9.6
Skåne	May 15	7	May 8	12.6

The listed statistics are mean values and standard deviations (SD) for the three study regions and the two different data interpretation cases, including the spatial variability assumption (SVA) and the observation bias assumption (OBA). Spatiotemporal statistics represent the full spatial and temporal variability of FF point data among all temperature grid cells (and for the SVA assumption all FF points within each grid cell) for each study area and over all years with FF data in the study period. Spatial statistics represent the spatial variability among the grid cells for their temporally averaged FF data over the whole period. Temporal statistics represent the temporal variability among the years in the study period of the spatially averaged FF data over each study area.

The species specificity is quantified in the FF(DD_C_) model through a temperature threshold (T_0_) that must be exceeded for the necessary insect development steps to take place toward the first flight. Whether and when the temperature threshold T_0_ is exceeded and the associated insect development steps occur during each year depends on the prevailing regional climate conditions. The latter are related to T_0_ through the calculation of daily degree-day contributions (ΔDD) to cumulative DD. One possible method for this calculation is as follows:

$$\begin{array}{c} \Delta DD=0\;if\;T_{\text{max}}\leq T_{0}\\ \Delta DD=T_{\text{mean}}-T_{0}\;if\;T_{\text{min}}\geq T_{0}\\ \Delta DD=0.5(T_{\text{max}}-T_{0})-0.25\left(T_{0}-T_{\text{min}}\right)\;if\;T_{\text{mean}}\geq T_{0}\;\&\;T_{\text{min}}<T_{0}\\ \Delta DD=0.25(T_{\text{max}}-T_{0})\;if\;T_{\text{mean}}<T_{0}\;\&\;T_{\text{max}}>T_{0} \end{array}$$ (1)

where T_max_, T_min_ and T_mean_ are the daily maximum, minimum and mean temperature values respectively, and T_mean_ is calculated as 0.5(T_max_+T_min_). This method for ΔDD calculation, which assigns a weighted value of ΔDD depending on the daily temperature values in each temperature grid cell, is reported by the UK Met Office [12] and is used here as one possible methodology for cumulative DD quantification.

More generally, the FF(DD_C_) modeling approach proposed here does not depend on which particular quantification method is used to calculate the cumulative DD. The novel, key aspect of the present approach is to test the possible existence and prediction capability of a species-specific, but climate- and region-independent DD_C_ value based on a set of relevant climate data, associated DD calculation results, and available observation data for FF (or some other DD-related phenological variable). Testing of the FF(DD_C_;T_0_) model and its key DD_C_ hypothesis regards then the model ability to robustly explain the observed spatiotemporal variability of FF within and across various regions and time periods of study.

Three study regions with availability of both temperature and FF observation data are then considered here, which fulfill requirements of relatively long-term data series and geographic region distribution along a steep climate gradient. Based on and across these regional and temporal data, we test DD_C_ values for various T_0_ scenarios. Specifically, we exemplify and illustrate DD_C_ test results for T_0_ = 0, 5.5 and 10°C in order to cover the model implications for a relatively wide range of possible T_0_ values of relevance, as reported from previous experimental studies in the literature [13].

For the DD_C_ testing, we calculate for each combination of T_0_ scenario and DD_C_ test value a model-implied result for FF(DD_C_;T_0_), as the date when the regional average cumulative DD of each considered year reaches the tested DD_C_ value. As a general look-up basis for finding the regional average date of DD_C_ achievement in each considered year, we first calculate cumulative DD (based on Eq (1)) for each temperature grid cell from the 1^st^ of January until every other day in each year of the period 1950–2010. We further average the cumulative DD cell values of each day across all temperature grid cells within each region, with S1 Dataset listing the resulting daily regional average DD values for each study region and year in the period 1950–2010, and each T_0_ scenario obtained by incrementing the T_0_ value by 0.5°C within the range 0°C ≤ T_0_ ≤ 10°C.

For the specific scenario examples of T_0_ = 0, 5.5 and 10°C and a range of DD_C_ test values, we further compare each associated FF(DD_C_;T_0_) model value with the corresponding regional average value of observed FF dates for each year. From this model-observation comparison, a best FF(DD_C_;T_0_) model for each region is determined as that minimizing the root mean square error (RMSE) relative to the corresponding observed regional FF across all years with available FF observation data.

Furthermore, the best regional model FF(DD_C_;T_0_) identified in this way is used to calculate model values of FF for each temperature grid cell and year, which are compared against all corresponding point observations of FF within each region, calculating also the RMSE resulting from this point observation comparison. The best regional models FF(DD_C_;T_0_) are further compared among the regions to quantify the ranges of best-model values of DD_C_ and T_0_ across all regions. We find these ranges to be quite narrow, thus supporting the existence of a climatically consistent species-specific DD_C_(T_0_) value. We base the evaluation of the latter on the mid-range value of DD_C_(T_0_) among the best regional models, and use the resulting cross-regional model FF(DD_C_;T_0_) to quantify the model-implied change in regional average FF due to observed climate change from the period 1951–1980 to the recent period 1981–2010.

## Results and Discussion

Considering all spatiotemporal data points (Fig 1B–1D and Fig 2A–2C), the investigation of the SVA case includes a total of 185 observations of FF distributed over 40 temperature grid cells (pixels) for the Medelpad/Ångermanland region, 906 observations over 33 grid cells for the Sörmland/Stockholm region, and 474 observations over 28 grid cells for the Skåne region. Considering only the earliest reported FF for each grid cell and year (white-circled points in Fig 1B–1D, with annual averages shown in Fig 2A–2C), the investigation of the OBA case includes a total of 101 observations for Medelpad/Ångermanland, 184 observations for Sörmland/Stockholm and 124 observations for Skåne.

The annual average FF date across all grid cells (Fig 2A–2C) as well as the total spatiotemporal average FF date across all grid cells and years (Fig 2D) is a few days later in the SVA case than in the OBA case, since the latter considers only the earliest annual FF sighting in each grid cell as the correct one for each year (see further summary statistics for each case listed in Table 1; S1 Fig shows additional details of the spatiotemporal variability of FF data). Average FF is further, for both assumption cases, delayed when going from south to north across the latitudinal climate gradient of mean annual temperature, which is 8.2°C in Skåne, 6.9°C in Sörmland/Stockholm and 3.6°C in Medelpad/Ångermanland (for the main FF data period 2003–2010).

**Fig 3:**
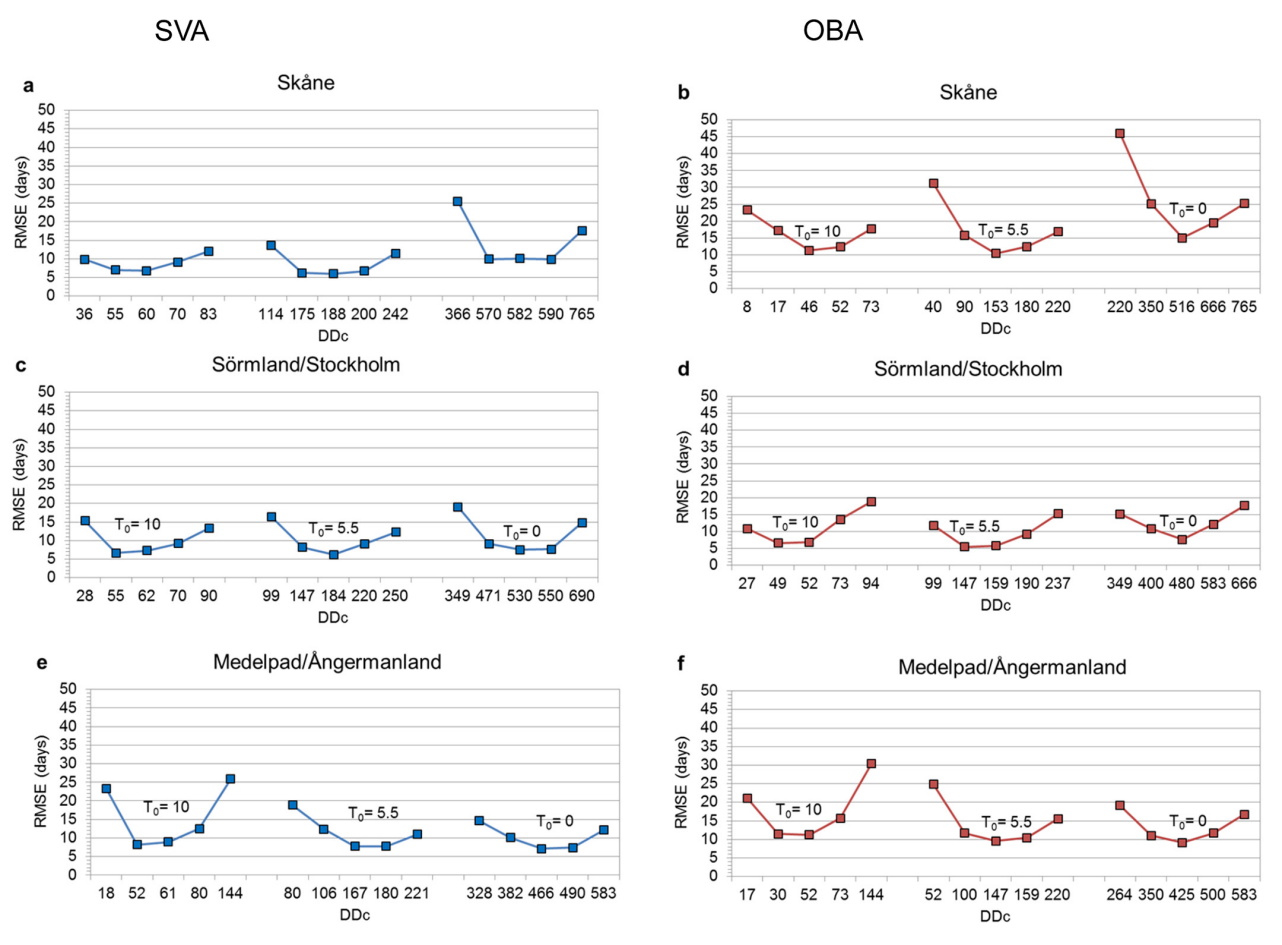
Root mean square error (RMSE) of modeled relative to observed first flight dates (FF). The RMSE results compare the modeled and observed regional average FF over all years with available FF data, for different temperature threshold, T_0_, and fixed value of cumulative degree-days, DD_C_, and for each study region: Skåne (a and b), Sörmland/Stockholm (c and d) and Medelpad/Ångermanland (e and f). Results are shown for the basic assumptions of spatial variability (SVA; left panels: a, c, e) and observation bias (OBA; right panels: b, d, f).

In the iterative testing of the FF(DD_C_;T_0_) model, we considered first a range of DD_C_ test values around and including the minimum, average and maximum value (among all years with FF data) of regional average cumulative DD (across all FF observation points in each region) as listed in S1 Dataset from the 1^st^ of January until the FF date of each observation point. For all exemplified T_0_ scenarios and all study regions, the DD_C_ value that minimizes the RMSE for regional average FF is found to actually fall within this primary range of DD_C_ test values (Fig 3). This finding indicates a relatively simple way to constraint a relevant DD_C_ test value range, which may be applicable and worthy of further testing across various regions and species.

Across the study regions, the most consistent FF(DD_C_;T_0_) model among those minimizing RMSE for each T_0_ scenario is identified as that for T_0_ = 5.5°C with associated DD_C_ = 167–188 for the SVA case (with resulting RMSE of 6–8 days) and DD_C_ = 147–159 for the OBA case (RMSE of 5–10 days). Even though the minimum RMSE for T_0_ = 5.5°C does not differ much from that for T_0_ = 0 or 10°C, the T_0_ = 5.5°C model is considered best because the associated DD_C_ values vary less among the different regions and assumption cases than those for T_0_ = 0 or 10°C (Fig 3). Specifically, for T_0_ = 5.5°C, the cross-regional range limits of the regionally best DD_C_ values are ±6% of the mid-range value for SVA, ±4% for OBA, and ±12% across both cases. Corresponding DD_C_ results for T_0_ = 0°C are 11%, 10% and 16%, while for T_0_ = 10°C they are 7%, 6% and 13%, respectively.

The predictive capability of the identified best model of regional average FF(DD_C_ = 147–188; T_0_ = 5.5°C) is further tested against all point observations of FF within each region. This test compares the point FF observation data with the results of the best regional model for each assumption case, SVA or OBA, and Table 2 lists the associated resulting RMSE. Under both the SVA and the OBA assumption, the point comparison yields a RMSE range of 13–16 days (Table 2), which is of similar magnitude as one standard deviation of the spatiotemporally variable point FF data (14–16 days, Table 1). The RMSE for the point test is, as expected, greater than the RMSE for regional average FF (of 5–10 days, Fig 3), since the latter is a spatial average that varies only in time. A two weeks error is also within the variability range reported in the literature for the advancement of butterfly appearance over a period of warming [2;3;4;14].

**Table 2:** Root mean square error (RMSE) in the point test of best regional models.

	RMSE (days)	
	Spatial variability assumption (SVA)	Observation bias assumption (OBA)
Medelpad/Ångermanland	16.1	15.7
Sörmland/Stockholm	13.4	13.7
Skåne	15	14.4

The calculated RMSE (Table 2) is relative to all spatiotemporal points of first flight (FF) observation for the best regional FF models: FF(DD_C_ = 167;T_0_ = 5.5°C) for Medelpad/Ångermanland, FF(DD_C_ = 184;T_0_ = 5.5°C) for Sörmland /Stockholm and FF(DD_C_ = 188;T_0_ = 5.5°C) for Skåne in the assumption case of spatial variability (SVA), and FF(DD_C_ = 147;T_0_ = 5.5°C), FF(DD_C_ = 159;T_0_ = 5.5°C) and FF(DD_C_ = 153;T_0_ = 5.5°C), respectively, in the assumption case of observation bias (OBA). The model parameters DD_C_ and T_0_ are the species-specific value of degree-day (DD) accumulation required until FF and the threshold temperature for DD calculation according to Eq (1), respectively.

**Fig 4:**
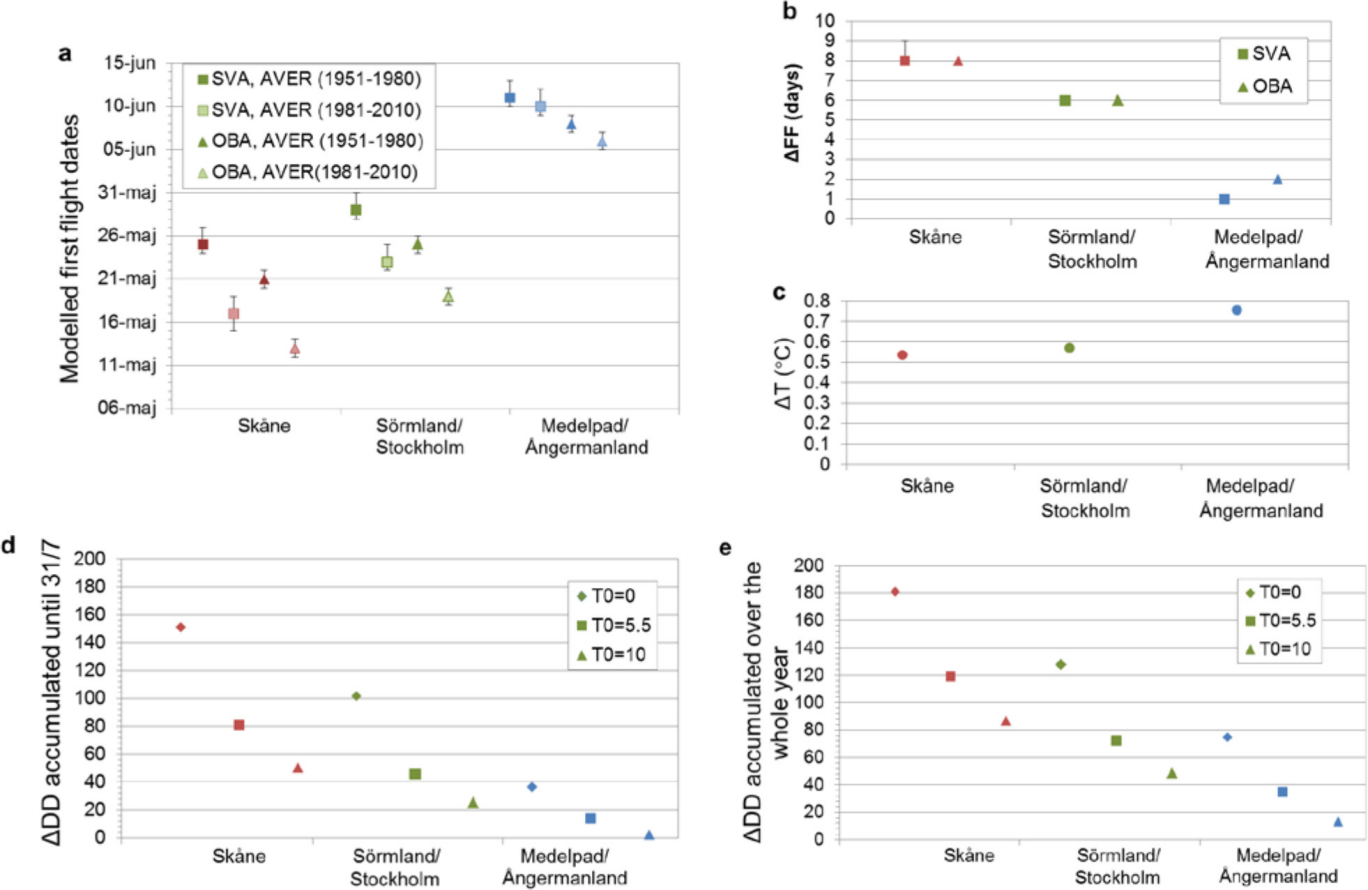
First-flight and climate change in average conditions from the period 1951–1980 to the period 1981–2010. a) Long-term regional average first-flight dates (FF) for the two periods and the three regions, as resulting from modeling with the temperature threshold T_0_ = 5.5°C and mid-range value of the species-specific degree-day constant DD_C_ = 177.5 for the spatial variability assumption (SVA) and DD_C_ = 153 for the observation bias assumption (OBA). Error bars quantify the range of modeled FF dates obtained by use of the range limits of best DD_C_ values. b) Modeled change in FF between the two periods (in terms of the number of days of FF advancement under warming) for each region and for the SVA and OBA cases. c) Change in regional average temperature between the two periods. d) Change between the two periods in regional average DD accumulated until 31/7 and e) over the whole year for each T_0_.

Model application to the observed historic warming in the study regions over the last 60 years demonstrates the model usefulness for assessing climate-driven FF change. For illustration of the results, we use here the mid-range best-model value of DD_C_ for each assumption case: FF(DD_C_ = 177.5;T_0_ = 5.5°C) for SVA and FF(DD_C_ = 153;T_0_ = 5.5°C) for OBA. Results quantify advancement of the long-term regional average FF dates (i.e., change to earlier FF dates) for both assumption cases SVA and OBA (Fig 4A and 4B) under the observed regional warming from 1951–1980 to 1981–2010 (Fig 4C). The spatial distribution of FF advancement among the regions (Fig 4B), however, is opposite to that of the mean temperature rise (Fig 4C) and instead consistent with that of the change in DD accumulated over some fixed annual time period, be it until mid-summer (Fig 4D) or over the whole year (Fig 4E); see further S2, S3 and S4 Figs for the whole time series of annual FF, temperature, and cumulative DD, respectively.

The reason for the found regional distribution of FF advancement is that the relatively small warming in the study region of Skåne (S3 Fig) has been most efficient in adding new daily contributions to the regional cumulative DD. This is because the mean regional temperature was and still is there sufficiently above the threshold temperature T_0_ for shifting relatively many daily temperature values from below to above T_0_. The larger warming in the two colder study regions has not been as efficient in this respect because the mean temperature was and still is there insufficiently higher than or too much below the threshold temperature T_0_.

Consideration of only mean temperature rise may thus mislead assessment of climate-driven FF change. Consideration of cumulative DD change over some reasonable fixed time, which is independent of FF but accounts for the species-dependent threshold temperature T_0_, is more useful for assessing the distribution of relative severity in FF change across different locations and regions. For quantification of actual FF change, account is also needed of the species-specific DD_C_ value that has to be accumulated for insect development to first flight.

The cross-regional consistency of the species-specific model FF(DD_C_;T_0_) developed in this study provides predictive capability for climate-driven FF change and its spatial distribution. This capability can be used for assessing future FF change under projected climate change scenarios, and should also apply to and needs further testing in other parts of the world. Furthermore, analogous modeling approaches can also be relevant for other species and phenological variables, and should therefore be developed and tested in further comparative investigations across different regions.

## Supporting Information

S1 Fig First flight dates (FF) and their average values for each grid cell with temperature data. Temperature grid cells are numbered sequentially from first row to the end and left to right in each region (Fig 1). Results are shown for the spatial variability assumption (SVA; left panels) and the observation bias assumption (OBA; right panels) for Skåne (a and b panels), Sörmland/Stockholm (c and d) and Medelpad/Ångermanland regions (e and f).
(TIF)

S2 Fig Dynamics and average values of modeled first flight dates (FF). Annual values of regional average FF are calculated for threshold temperature T_0_ = 5.5°C and associated constant degree day values DD_C_ = 177.5 for the spatial variability assumption (SVA) and DD_C_ = 153 for the observation bias assumption (OBA). Average values are shown for the two 30-years periods 1951–1980 and 1981–2010.
(TIF)

S3 Fig Annual average temperature and long-term 30-years average temperature for the two periods: 1951–1980 and 1981–2010. (TIF)

S4 Fig Regional average degree days (DD) accumulated over the whole year for threshold temperature: a) T_0_ = 0°C; b) T_0_ = 5.5°C and c) T_0_ = 10°C. (TIF)

S1 Dataset Regional average cumulative degree days for different temperature threshold values for the period 1950–2010. (ZIP)
