# Supplementary figures and images for: Climate-Driven Phenological Change: Developing Robust Spatiotemporal Modeling and Projection Capability
Source: PLoS One. 2015 Nov 6;10(11):e0141207. doi: 10.1371/journal.pone.0141207 (PMC4636262; doi:10.1371/journal.pone.0141207)

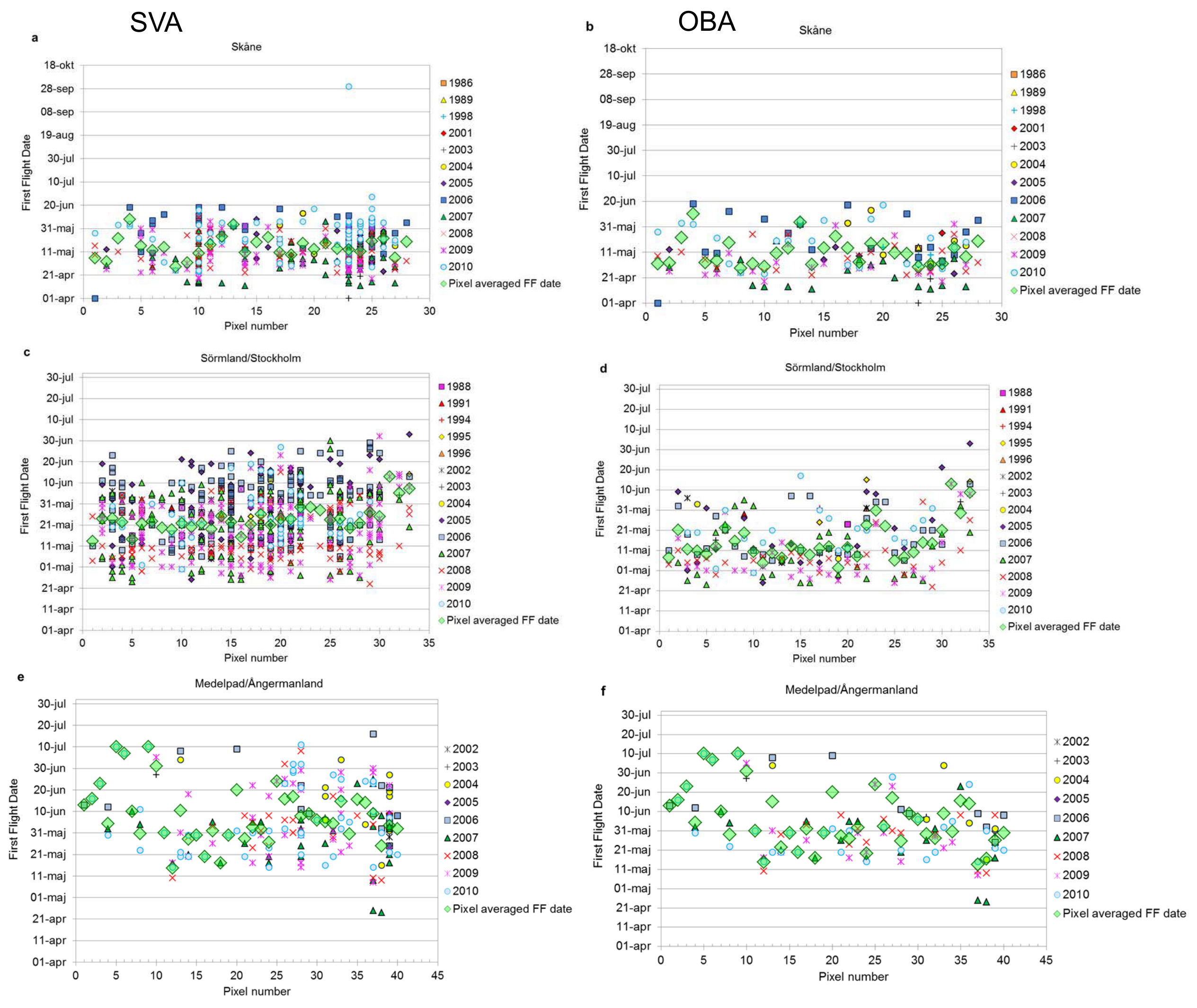

Supplement: S1 Fig — Temperature grid cells are numbered sequentially from first row to the end and left to right in each region (Fig 1). Results are shown for the spatial variability assumption (SVA; left panels) and the observation bias assumption (OBA; right panels) for Skåne (a and b panels), Sörmland/Stockholm (c and d) and Medelpad/Ångermanland regions (e and f). (TIF) [file pone.0141207.s001.tif]

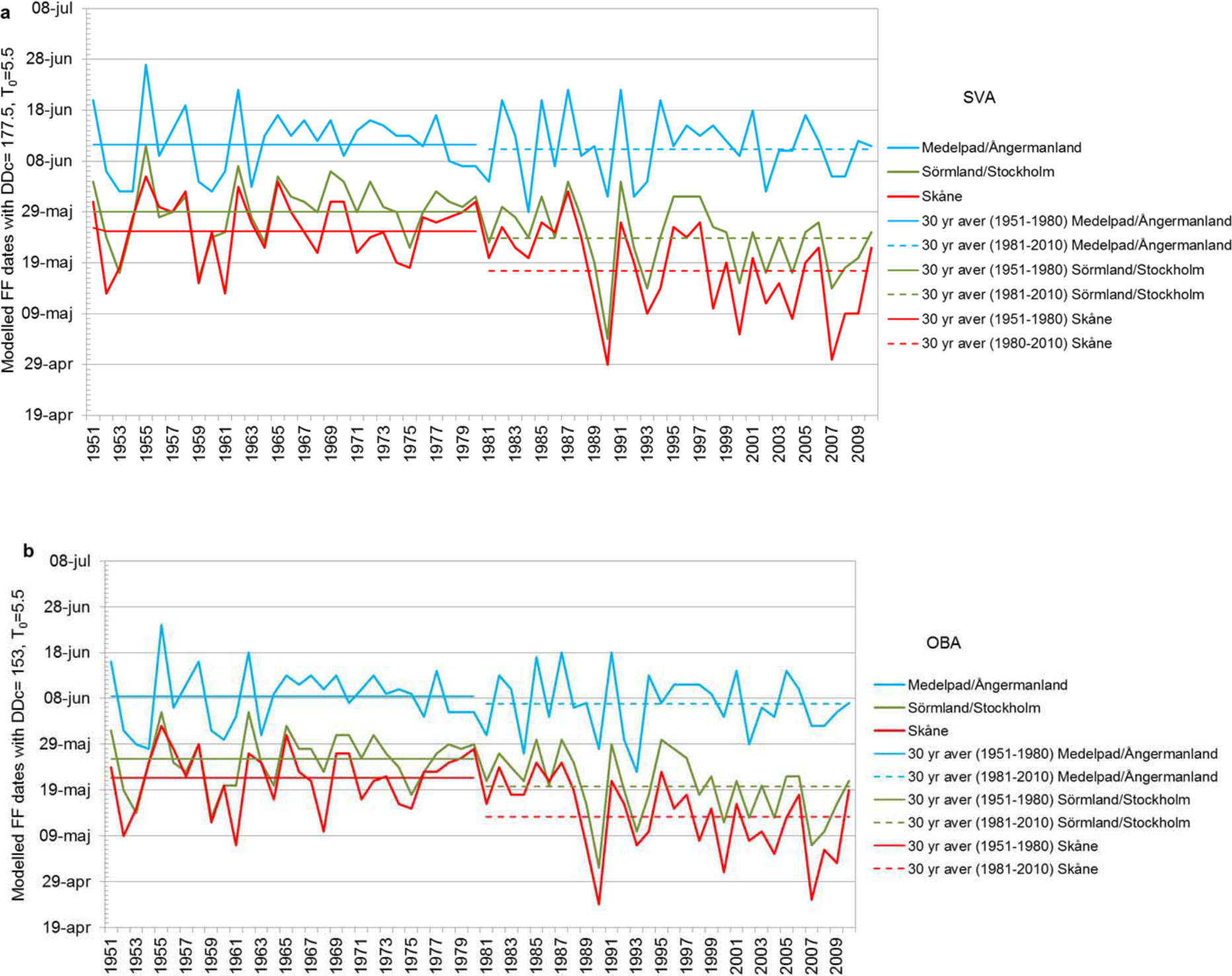

Supplement: S2 Fig — Annual values of regional average FF are calculated for threshold temperature T0 = 5.5°C and associated constant degree day values DDC = 177.5 for the spatial variability assumption (SVA) and DDC = 153 for the observation bias assumption (OBA). Average values are shown for the two 30-years periods 1951–1980 and 1981–2010. (TIF) [file pone.0141207.s002.tif]

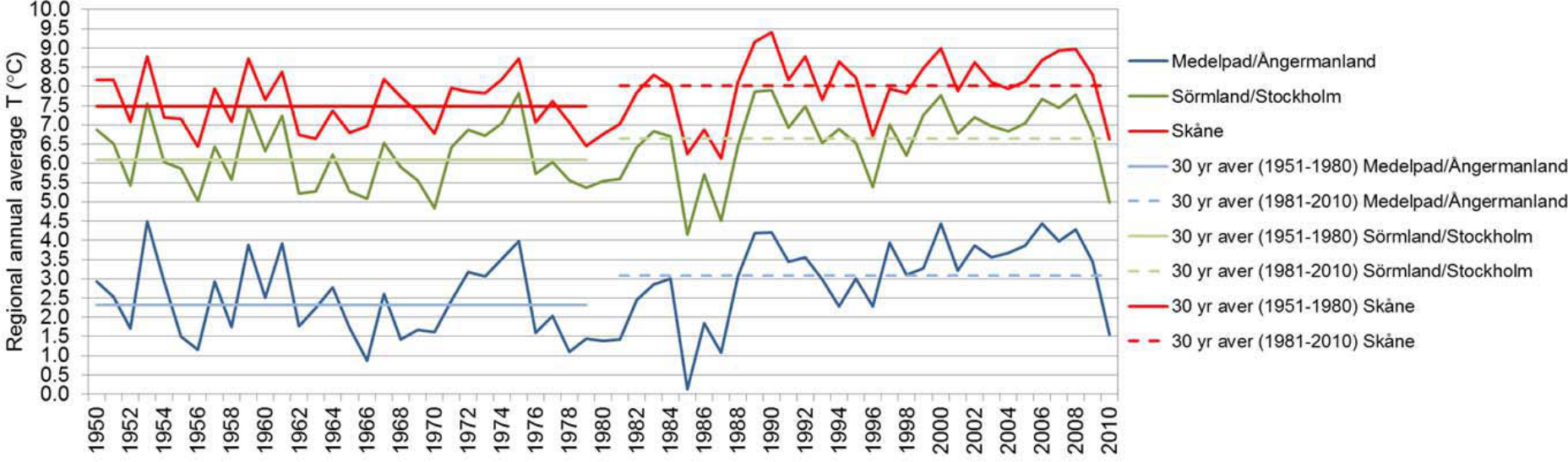

Supplement: S3 Fig — (TIF) [file pone.0141207.s003.tif]

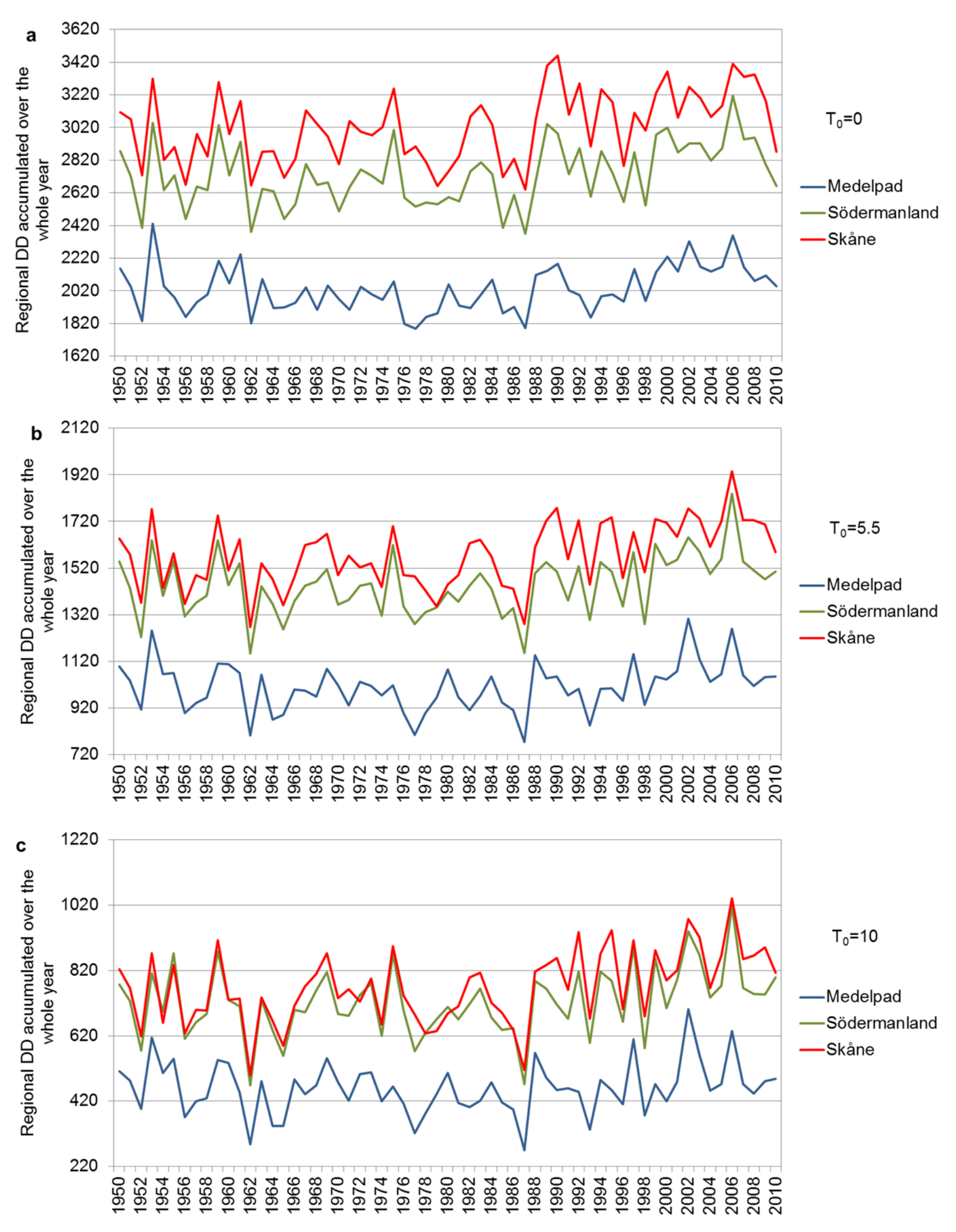

Supplement: S4 Fig — (TIF) [file pone.0141207.s004.tif]
